# All hands on deck: Malaria control urgently needs workable public-private-philanthropic partnerships (PPPP)

**DOI:** 10.1371/journal.pgph.0001891

**Published:** 2023-04-25

**Authors:** Kolawole Maxwell, Elisabeth G. Chestnutt, Perpetua Uhomoibhi, Christian Kompaoré, Jimmy Opigo, James K. Tibenderana

**Affiliations:** 1 Malaria Consortium, Abuja, Nigeria; 2 London School of Hygiene and Tropical Medicine, London, United Kingdom; 3 Malaria Consortium, London, United Kingdom; 4 National Malaria Elimination Programme, Abuja, Nigeria; 5 Programme National de Lutte Contre le Paludisme, Burkina Faso; 6 National Malaria Control Programme, Uganda; PLOS Global Public Health and APHRC, KENYA

Between 2000 and 2015, great progress was made to drive down malaria, with incidence and mortality rates from the disease falling by 27% and 50% respectively [1]. This was a result of effective programming and the increased global attention and funding for the disease. However, since 2015 the mortality rate has fallen by less than 2% and the funding gap has continued to widen from US$ 2.6 billion in 2019 to US$ 3.8 billion in 2021 [1]. The COVID-19 pandemic further pushed back progress, especially in sub-Saharan Africa where malaria cases rose by over 7% between 2019 and 2021 while funding remained stagnant [1]. For the malaria community to tip the balance back towards eradication an all hands on deck approach must be taken. We propose creating the conditions that allow public-private-philanthropic (PPP) partnerships i.e. collaborations between public, private for-profit, and private non-profit health sub-sectors, to help sustain the progress towards malaria eradication in Africa.

The private sector for health, even for malaria services, is well developed in Sub-Saharan Africa; in countries such as Nigeria and Uganda, over half of citizens receive treatment for malaria in the private sector [2, 3]. A large number of citizens also purchase malaria commodities from private vendors and pharmacies [2, 3]. However, the private sector is largely unregulated, and the type, quality, and affordability of services vary. Because available products and services are often determined by customer demand, patients may not always have access to products such as rapid diagnostic tests (RDTs) or quality-assured (QA) medicines which are often seen as an unnecessary additional expense. In the public sector RDT coverage is on average 64% whereas in the formal private sector it is just 48% [1]. A lack of testing availability can lead to the unnecessary prescription and use of antimalarials, since presumptive diagnosis of malaria can be incorrect up to 90% of the time [4], and increases the risk of antimalarial drug resistance.

Attempts have been made to improve the quality and affordability of services in the private sector through public-private partnerships such as the Affordable Medicines Facility for Malaria (AMF-m) which transitioned to the Private Sector Co-payment Mechanism (PSCM) and Unitaid’s Private Sector Malaria RDT project. The AMF-m significantly increased the percentage of private for-profit outlets with availability of QA Artemisinin-based combination therapies (ACTs) in five countries (Ghana from 25% to 83%, Kenya from 21% to 60%, Nigeria from 27% to 53% and Tanzania from 11% to 66%) [5]. However further studies are needed to assess the long-term sustainability of the results since the partnership ended in 2017. Similarly, Unitaid’s Private Sector Malaria RDT project aimed to promote parasitological diagnosis in the private sector. The project had some success at increasing availability of RDTs in the private sector but it was challenging to reduce the price enough to create sustainable demand. The project concluded in 2016 and noted that if no additional market development activities take place, these markets will either remain at the same levels or reverse [6].

While there is limited data following the conclusion of these projects, relying on subsidies for market shaping is unlikely to be sustainable. Instead, PPP partnerships may provide the sustainability that donor-funded programs lack and supports progress towards malaria elimination and universal health coverage, as demonstrated in Fig 1.

**Fig 1:**
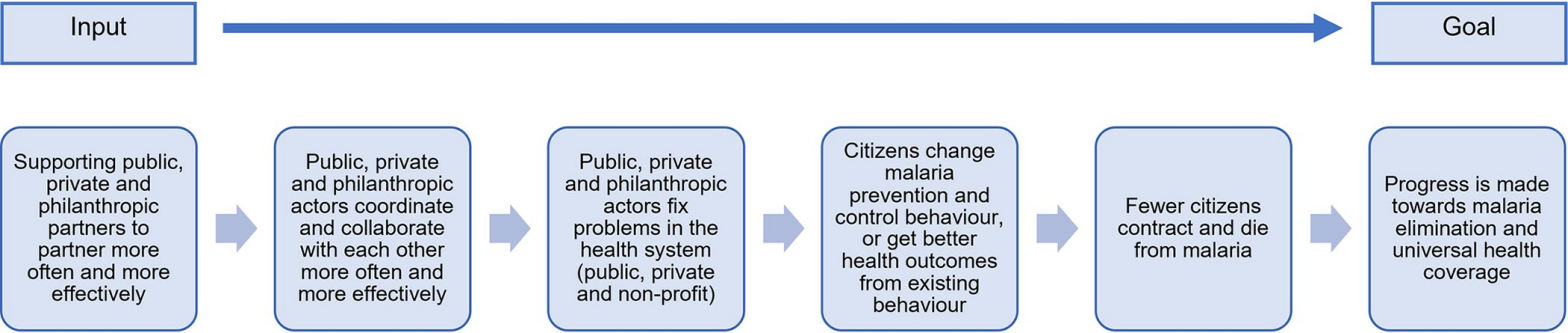
The results chain framework for public-private-philanthropic partnerships.

Facilitating these partnerships in Nigeria has shown promising results. During the Support to the National Malaria Programme in Nigeria project, partnerships were formed between professional associations and business member-based organisations to strengthen commercial retail of long-lasting insecticide-treated nets (LLIN) in Nigeria. An initial analysis found that medical professionals and informal drug sellers were not providing information to customers on the need to purchase and use LLIN. In some cases the providers themselves lacked knowledge of these products and their benefits, which meant they were unable to inform customers or patients.

Two partnerships were designed to promote LLIN through the pharmacists’ network and provide training to medical staff and vendors on LLIN’s and their use. This training led to a greater understanding of the value of LLIN and the importance of promoting them and, as a result, 60% of medical professionals began prescribing LLIN after the training [7]. Pharmacists also increased LLIN promotion, 88% increased dialogue around LLIN with customers and 63% added promotional materials to increase sales [7]. These activities created a market response where 70% of pharmacists started to stock LLIN and 50% reported an increase in sales of LLIN [7]. Inspired by these results, the implementing partner organised additional workshops and refresher trainings without financial support.

Building on this experience, we propose a framework that facilitates actors to collaborate more effectively by leveraging their mutual interests. The framework sets out four overarching steps that should be taken to facilitate sustainable PPP partnerships. First, a shared understanding of PPP partnerships is necessary for the actors to recognise the interconnected environment and that each health sub-sector cannot achieve their goals alone. This will create incentives for partnership and encourage identification of opportunities that could enhance progress towards shared goals and sustainability. In Ethiopia, a memorandum of understanding was used to provide a formal agreement for a public-private partnership which aimed to improve malaria case management, however informal methods of governance can also be used if PPP partnerships are coordinated at the correct level mandated in each country’s health system [8]. Next, to ensure they are able to facilitate and support these partnerships, the Minister of Health must have a more inclusive planning process which involves private and philanthropic actors from the beginning. This is being implemented in Nigeria as part of the National Development Plan 2021–2025 [9]. However, existing private sector partners must not be the only ones to benefit from these incentives and the government must extend this to new providers to create competition. This could reduce the price of commodities, raise the quality of services and support the provision of sustainable high-quality health care. There are often tensions between commercial pressures and public health goods, therefore the government must recognise their governance role in managing PPP partnerships and supporting partners to navigate competing priorities. Government-led incentives may be required, these could include training opportunities for private sector health providers or strengthening the enforcement of regulations. Finally, creating a platform for consistent collaboration and information sharing will help actors to see first-hand the benefits of partnership. For example, integrating data from private health facilities will help the national malaria programme to build a full picture of the malaria situation. In return, the private sector could benefit from training or tools to facilitate data collection and sharing. These factors are critical for resolving cross-cutting issues, such as mistrust between actors and a lack of capacity, which are currently limiting success.

Building capacity within national and sub-national governments to identify opportunities and facilitate partnerships is critical for PPP partnerships to flourish. Once incentives and responsibilities have been defined these partnerships can be self-sustaining. Partnerships between actors within each sector can increase efficiencies and reduce duplication while collaborations between health sub-sectors can provide greater overall impact and sustainability. This approach also strengthens country ownership and can be applied to integrate multiple-diseases programmes within countries and to support global progress towards achieving the Sustainable Development Goals. Adopting this way of working, in a way that is tailored to each national and subnational context, will increase access to QA malaria commodities and in turn drive down cases and save numerous lives. Continuing a business-as-usual approach, which isolates critical actors, will not be sufficient to eradicate malaria and, with current constraints on resources, will lead to more lives being lost unnecessarily.

## References

[1] World Health Organization. World malaria report 2022. Geneva, Switzerland: World Health Organization; 2022.

[2] National Malaria Elimination Programme (NMEP) [Nigeria], National Population Commission (NPC) [Nigeria], and ICF. Nigeria Malaria Indicator Survey 2021 Final Report. Abuja, Nigeria, and Rockville, Maryland, USA: NMEP, NPC, and ICF; 2022.

[3] Uganda National Malaria Control Division (NMCD), Uganda Bureau of Statistics (UBOS), and ICF. Uganda Malaria Indicator Survey 2018–19. Kampala, Uganda, and Rockville, Maryland, USA: NMCD, UBOS, and ICF; 2020.

[4] Ladner J., Davis B., Audureau E. and Saba J. Treatment-seeking patterns for malaria in pharmacies in five sub-Saharan African countries. Malaria Journal. 16(1):1–3. 2017. doi: 10.1186/s12936-017-1997-3 28851358 PMC5574241

[5] AMFm Independent Evaluation Team. Independent Evaluation of Phase 1 of the Affordable Medicines Facility—malaria (AMFm), Multi-Country Independent Evaluation Report: Final Report. Calverton, Maryland, USA and London, UK: ICF International and London School of Hygiene and Tropical Medicine; 2012.

[6] Dalberg. Unitaid end of project evaluation: Creating a private sector market for quality-assured mRDTs. https://unitaid.org/assets/20170224_mRDT-private-sector-market-Final-evaluation_FINAL.pdf [accessed January 2023].

[7] Montrose and Innovision. Case study: Strengthening commercial retail of LLIN through partnership with professional associations and business member-based organisations: The SuNMaP Nigeria Experience. 2014. https://www.malariaconsortium.org/media-downloads/1678/Strengthening%20commercial%20retail%20of%20LLINs%20through%20partnership%20with%20professional%20associations%20and%20business%20member-based%20organisations [Accessed April 2023].

[8] Argaw M.D., Woldegiorgis A.G., Abate D.T. and Abebe M. E. Improved malaria case management in formal private sector through public private partnership in Ethiopia: retrospective descriptive study. Malaria Journal. 15(352). 2016. doi: 10.1186/s12936-016-1402-7 27401095 PMC4940756

[9] Federal Ministry of Finance, Budget and National Planning [Nigeria]. National Development Plan (NDP) 2021–2025 Volume I. https://nationalplanning.gov.ng/wp-content/uploads/2021/12/NDP-2021-2025_AA_FINAL_PRINTING.pdf [accessed April 2023].

